# Supplementary material for: Systematic review of instruments for assessing culinary skills in adults: What is the quality of their psychometric properties?
Source: PLoS One. 2021 Aug 9;16(8):e0235182. doi: 10.1371/journal.pone.0235182 (PMC8351978; doi:10.1371/journal.pone.0235182)
Supplement: S2 Table — (PDF) [file pone.0235182.s004.pdf]

S2 Table. List of excluded studies.

Reason for exclusion	Author, Year	Title	Reference
Wrong outcome	Bech Larsen & Tsalis, 2018	Impact of cooking competence on satisfaction with food-related life: Construction and validation of cumulative experience & knowledge scales	Food Quality and Preference. 68: 191–197. doi: 10.1016/j.foodqual.2018.02.006
Wrong outcome	Bongoni, et.al , 2015	Evaluation of research methods to study domestic food preparation	British Food Journal. 117(1):7-21 doi: 10.1108/BFJ-09-2013-0273
Wrong outcome	Garaham et. al, 2013	Perceived Social Ecological Factors Associated with Fruit and Vegetable Purchasing, Preparation, and Consumption among Young Adults	Journal of the American Academy of Nutrition and Dietetics. 113(10) doi:10.1016/j.jand.2013.06.348
Wrong outcome	Ko, 2010	To Evaluate the Professional Culinary Competence of Hospitality Students	Journal of Culinary Science & Technology. 8(2):136-146 doi: 10.1080/15428052.2010.511101
Wrong outcome	Lane, et. al, 2017	Development of the Cooking and Food Provisioning Action Scale (CAFPAS): A new measurement tool for individual cooking practice	Food Quality and Preference. 62(2) doi: 10.1016/j.foodqual.2017.06.022
Wrong outcome	Pinard et. al, 2018	Development and Testing of a Revised Cooking Matters for Adults Survey	American Journal of Health Behavior. 39(6):866-873 doi: 10.5993/AJHB.39.6.14
Wrong outcome	Poncet et. al, 2015	Reliability of the Cooking Task in adults with acquired brain injury	Neuropsychological Rehabilitation. 25(2):298-317. doi: 10.1080/09602011.2014.971819.
